# Altered putamen functional connectivity is associated with anxiety disorder in Parkinson's disease

**DOI:** 10.18632/oncotarget.18996

**Published:** 2017-07-05

**Authors:** Xixi Wang, Junyi Li, Yongsheng Yuan, Min Wang, Jian Ding, Jiejin Zhang, Lin Zhu, Yuting Shen, Hui Zhang, Kezhong Zhang

**Affiliations:** ^1^ Department of Neurology, The First Affiliated Hospital of Nanjing Medical University, Nanjing, China; ^2^ Department of Radiology, The First Affiliated Hospital of Nanjing Medical University, Nanjing, China

**Keywords:** Parkinson’s disease, anxiety, resting state functional magnetic resonance imaging, FC, pathogenesis

## Abstract

In this study, we used resting state-functional magnetic resonance imaging (rs-fMRI) to explore altered putamen functional connectivity (FC) in Parkinson's disease patients with anxiety disorder. We divided 65 Parkinson's disease patients into anxiety (PD-A; *n*=18) and non-anxiety (PD-NA; *n*=45) groups based on a Hamilton Anxiety Rating Scale cutoff score of 12. The PD-A patients exhibited altered putamen FC with cortical and subcortical regions. The PD-A patients showed enhanced putamen FC with the caudatum, which correlated with increased emotional processing during anxiety. Decreased putamen FC with the orbitofrontal gyrus and cerebellum also correlated with increased anxiety in Parkinson's disease. Our findings demonstrate that anxiety disorder in Parkinson's disease is associated with abnormal putamen FC networks, especially with caudatum, orbitofrontal gyrus and cerebellum.

## INTRODUCTION

Anxiety syndrome is more prevalent in Parkinson's disease (PD) patients (6%-55%) [1] than in the general population and non-PD patients [2, 3]. Furthermore, anxiety in PD is associated with increased motor symptoms and negatively impacts quality of life [2]. Anxiety is clinically under-diagnosed in PD patients and is neglected while researching PD pathology.

In recent years, neuroimaging technology has been widely used to investigate functional and structural alterations of motor and non-motor symptoms in PD brains [4, 5]. Positron emission computed tomography (PET), single photon emission computed tomography (SPECT) and T1 structural magnetic resonance imaging (MRI) have been used to image individual brain regions while investigating anxiety disorders in PD [4]. Most PET/SPECT reports show correlation between putamen and severity of anxiety [6–9]. However, the association between anxiety in PD and alterations in putamen FCfunctional connectivity (FC) with other brain regions is unknown.

Resting state-functional MRI (rs-fMRI) measures the resting blood oxygen level-dependent signal to determine spontaneous brain activity between functionally linked brain regions at the level of neural networks. FC indicates inter-regional temporal patterns of the blood oxygen level-dependent signal [10]. Recently, rs-fMRI has been extensively used to explore neurological and psychiatric diseases [11, 12], including various motor [13]and non-motor symptoms [14–16] in PD.

The aim of this study was to use rs-fMRI to analyze differences in the putamen FC patterns among 3 study subject groups:, namely, PD patients with anxiety (PD-A), PD patients without anxiety (PD-NA) and healthy controls.

## MATERIALS AND METHODS

### Study subjects

Sixty-five right-handed PD patients were recruited from the outpatient clinic of our hospital and diagnosed with idiopathic PD according to the UK Parkinson's Disease Society Brain Bank criteria [17]. The following patients were excluded from the study: (1) patients with corticobasal degeneration, progressive supranuclear palsy, multiple system atrophy, vascular parkinsonism, and other forms of parkinsonism; (2) patients with contraindications to MRI such as claustrophobia, metallic implants or devices in the body; (3) patients with severe cognitive decline indicated by Mini Mental State Examination (MMSE) score < 24; (4) patients with clinically prominent depressive symptoms (17-item Hamilton Depression Rating Scale, HAMD score > 14) for controlling possible hybrid factors. None of the patients took anti-psychotic drugs. MRI scans and clinical examinations were performed at least 12 h after withdrawal from medications to mitigate the pharmacological effects on neural activity. Meanwhile, 24 healthy controls without psychological and neurological disturbances or neuroimaging abnormalities were recruited for this study. Written informed consent was obtained from all participants. The study was approved by the ethics committee of The First Affiliated Hospital of Nanjing Medical University.

### Clinical assessment of study subjects

PD patients were classified into PD-A (*n* = 18) and PD-NA (*n* = 45) groups based on the cut off score of 12 on the Hamilton Anxiety Rating Scale (HAMA) score [18–20]. The HAMA scale is a fourteen-item scale (total scores ranging 0-56) that measures both psychic and somatic anxiety symptoms [18], which is useful in evaluating anxiety severity in clinical practice and research [19]. Anxiety disorders of PD patients were assessed by a trained movement disorder specialist (Kezhong Zhang), sensitized to psychiatric disorders in PD. The Unified Parkinson's Disease Rating Scale (UPDRS-III) and Hoehn & Yahr (H&Y) staging scales were used to assess PD motor severity and disease stage, respectively. Total levodopa-equivalent daily dose (LEDD), LEDD of levodopa preparations and LEDD of dopamine receptor agonists of each PD patient were calculated as previously described [21]. Disease duration of PD patients and education levels of all subjects were recorded. Cognitive function, executive function and symptoms of depression were also quantified separately by MMSE, Frontal Assessment Battery (FAB) and HAMD.

### Rs-fMRI methodology

MRI scanning were performed with a 3.0 T Siemens MAGNETOM Verio whole-body MRI system (Siemens Medical Solutions, Germany) equipped with eight-channel, phase-array head coils. Tight foam padding was used to minimize head movement and ear-plugs were used to reduce noise. Subjects were instructed to remain motionless, close their eyes, remain awake, and not to think about anything in particular. T1-weighted 3D high resolution anatomical images were acquired using the following volumetric 3D magnetization-prepared rapid gradient-echo (MP-RAGE) sequence: repetition time (TR) = 1900 ms, echo time (TE) = 2.95 ms, flip angle (FA) = 9°, slice thickness = 1 mm, slices = 160, field of view (FOV) = 230 × 230 mm^2^, matrix size = 256 × 256 and voxel size = 1 × 1× 1 mm^3^. Resting-state functional images were collected using the following echo-planar imaging (EPI) sequence: TR = 2000 ms, TE = 21 ms, FA = 90°, FOV = 256 × 256 mm^2^, in-plane matrix = 64 × 64, slices = 35, slice thickness = 3 mm, no slice gap, voxel size = 3 × 3 × 3 mm^3^, total 4 volumes = 240.

### Rs-fMRI data analysis

The rs-fMRI data was analyzed by the data processing assistant for resting-state fMRI (DPARSF, http://www.restfmri.net/forum/dparsf) [22] with Statistical Parametric Mapping (SPM8, http://www.fil.ion.ucl.ac.uk). The analysis included 1) removal of the first 10 time points; 2) slice timing correction; 3) head motion correction *via* six-parameter rigid body spatial transformation during data acquisition; 4) nonlinear registration of the high-resolution T1 structural images to the Montreal Neurological Institute (MNI) template and segmenting them into white matter, gray matter, and cerebrospinal fluid using the DARTEL (diffeomorphic anatomical registration through exponentiated lie algebra) algorithm [23] followed by further structural analyses of the resulting segments; 5) nuisance signal removal (white matter, cerebrospinal fluid, global signal, 6-head motion parameters as covariates) *via* multiple regression; 6) spatial normalization to the Montreal Neurological Institute template; 7) resampling images into a spatial resolution of 3×3×3 mm^3^; 8) spatial smoothening with a Gaussian kernel (full width at half-maximum = 4×4×4 mm^3^); 9) temporal bandpass filtering (0.01 < f < 0.08 Hz) and linear detrended removal. We excluded subjects from further analysis if the translation or rotation of head movement was > 2 mm or 2° in any direction. Additionally, the mean head translation, mean head rotation, and frame-wise displacement were calculated [24]. There were no differences in head motion parameters among the three groups (*p* > 0.05).

### Functional connectivity analysis

The left and right putamen were defined as two regions of interest (ROI) based on the Anatomical Automatic Labeling (AAL) template by selecting specific areas to study anxiety-dependent resting-state functional networks in PD patients. A voxel-wise FC analysis was performed by computing the temporal correlation between the mean time series of each ROI and the time series of each voxel within the brain. Pearson's correlation coefficient maps were created for each individual subject and were converted to a z-value using Fisher's z transformation.

Analysis of covariance (ANCOVA) was performed to identify brain areas with differences between the three study subject groups with age, gender, education, HAMD scores and gray matter volume as covariates (voxel-level *p* < 0.05; cluster size > 113 voxels; corrected *p* < 0.01 as determined by AlphaSim correction). These areas were then extracted as a mask. Then, two-sample post hoc t tests were performed within this mask, with age, gender, education, HAMD scores and gray matter volume as covariates, to detect differences between study subject groups (voxel-level *p* < 0.05; cluster size of the left putamen > 13 voxels; cluster size of the right putamen > 16 voxels; corrected *p* < 0.01 as determined by AlphaSim correction). Additionally, for each ROI, the clusters that showed differences in FC between PD-A and PD-NA groups were extracted separately. Then the average FC value of each cluster was calculated to estimate the correlation of anxiety severity. The Pearson correlation between mean FC values and HAMA scores was calculated by IBM SPSS statistics v20.0.0 software (SPSS, Chicago, IL, USA) and the significance was set at *p* < 0.01 (two-tailed; http://afni.nimh.nih.gov/pub/dist/doc/manual/AlphaSim.pdf).

### Statistical analysis

The clinical data was analyzed with the IBM SPSS statistics v20.0.0 software and expressed as mean ± s.d. The continuous and categorical variables were analyzed by either one-way analysis of variance test, chi-square test, independent-sample *t*-test or nonparametric tests. *p* < 0.05 was considered statistically significant.

## RESULTS

### Demographic and clinical characteristics

The demographic and clinical characteristics of PD-A, PD-NA and healthy control subjects are summarized in Table 1. The three subject groups showed differences in age, gender, HAMD scores, but education levels were similar. The PD-A and PD-NA subjects had similar disease duration, H&Y staging, total LEDD, LEDD of levodopa preparations and LEDD of dopamine receptor agonists and UPDRSIII scores (*p* > 0.01). The MMSE and FAB scores for PD-A and PD-NA subjects was also similar. As expected, the HAMA scores were different between the three groups (*p* < 0.01).

**Table 1:** Demographic and clinical characteristics of all subjects

Groups	PD-A(*n*=18)	PD-NA(*n*=45)	HC(*n*=24)	*P* value
Gender (M/F)	12, 6	34, 11	10,14	0.02^a^
Age (y)	71.74±5.16	66.17±8.11	65.33±4.65	0.01^b^
Education (y)	12.84±2.95	11.52±3.56	10.79±2.92	0.13^b^
Disease duration (y)	3.76±3.23	3.94±2.87	NA	0.83^c^
H&Y staging	2.45±0.60	2.11±0.71	NA	0.14^c^
UPDRS-III	24.05±8.92	21.52±10.59	NA	0.36^c^
Total LEDD (mg/d)	450.17±252.08	373.95±306.93	NA	0.34^c^
LEDD of DA (mg/d)	33.55±43.91	40.90±40.95	NA	0.52^c^
LEDD of LP (mg/d)	346.05±156.85	257.32±223.62	NA	0.13^c^
MMSE	27.79±1.87	28.24±1.57	NA	0.32^c^
FAB	27.79±1.87	28.24±1.57	NA	0.32^c^
HAMD	9.26±2.64	3.83±3.19	1.54±2.06	<0.001^b^
HAMA	15.47±3.01	5.93±3.42	2.33±2.04	<0.001^b^

Data are presented as mean ± s.d..

Abbreviations: PD-A, Parkinson's disease with anxiety; PD-NA, Parkinson's disease without anxiety; HC, Healthy controls; NA, Not applicable; M, Male; F, Female; y, year; H&Y, Hoehn and Yahr; LEDD, Levodopa equivalent daily dose; DA, Dopamine receptor agonists; LP, Levodopa preparations; UPDRS, Unified Parkinson's Disease Rating Scale; MMSE, Mini Mental State Examination; FAB, Frontal Assessment Battery; HAMD, 17-item Hamilton Depression Rating Scale; HAMA, Hamilton Anxiety Rating Scale.

*P* < 0.05 was considered significant. ^a^chi-square test; ^b^one-way analysis of variance test (ANOVA); ^c^two-sample t-test.

### Functional connectivity data

There were differences in the left putamen FC with right orbitofrontal gyrus, left middle frontal gyrus, right postcentral cortex and left anterior cingulate among all three study groups, namely, PD-A, PD-NA and healthy controls. Similarly, all three study groups showed differences in right putamen FC with the left orbitofrontal gyrus, left middle frontal gyrus, bilateral paracentral lobule, right precuneus, left temporal pole, left middle occipital gyrus, right cerebellum, right insula, bilateral middle cingulate gyrus and left caudatum.

**Figure 1:**
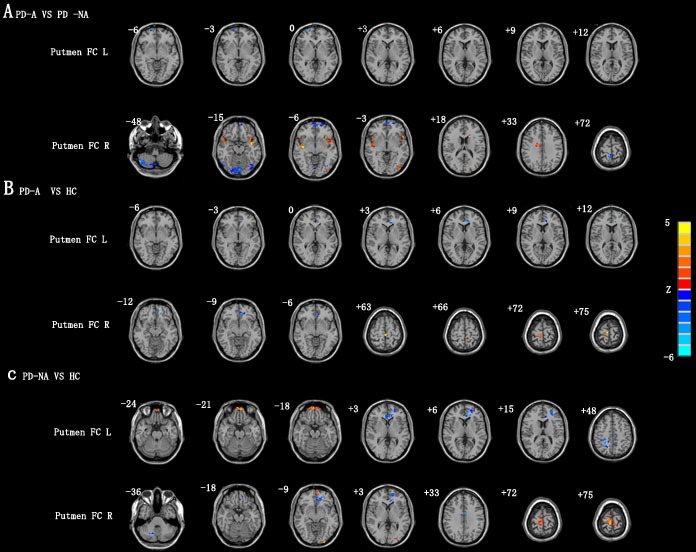
Analysis of putamen FC differences in PD-A and PD-NA patients **A**. Comparison of putamen FC in PD-A and PD-NA patients. **B**. Comparison of putamen FC in PD and healthy control subjects. **C**. Comparison of putamen FC in PD-NA and healthy control subjects. Note: *p* < 0.01 corrected by AlphaSim denoted statistical significance. HC, healthy control; PD-A, Parkinson’s disease patients with anxiety; PD-NA, Parkinson’s disease patients without anxiety.

Next, two-sample post hoc *t*-tests were performed to detect pair-wise differences in FC of the putamen regions among the PD-A, PD-NA and healthy control groups. The PD-A group showed following differences with PD-NA group: (1) reduced left putamen FC with right orbitofrontal gyrus; (2) reduced right putamen FC with left orbitofrontal gyrus, right cerebellum and right precuneus; (3) increased right putamen FC with right insula, left temporal pole, left middle occipital gyrus, left caudatum and right middle cingulate gyrus (Table 2 and Figure 1).

**Table 2:** Pair-wise differences in the putamen FC

Brain regions	Side	Cluster size (mm^3^)	Coordinates MINI			Z value
			X	Y	Z	
PD-A > PD-NA						
Right putamen FC						
Insula	R	254	39	-9	-6	4.713
Temporal pole	L	167	-39	-3	-15	4.343
Middle occipital gyrus	L	25	-42	-87	-3	3.162
Caudate	L	33	-15	15	18	3.976
Middle cingulate gyrus	R	36	12	-6	33	3.208
PD-A < PD-NA						
Left putamen FC						
Orbitofrontal gyrus	R	13	18	60	3	-3.130
Right putamen FC						
Cerebellum	R	744	51	-63	-48	-5.199
Orbitofrontal gyrus	L	143	-6	63	-3	-3.744
Precuneus	R	26	0	-45	72	-3.981
PD-A > HC						
Right putamen FC						
Paracentral lobule	L	20	0	-30	63	3.755
Paracentral lobule	R	21	6	-24	75	3.590
PD-A < HC						
Left putamen FC						
Anterior cingulate gyrus	L	35	-12	36	3	-4.136
Right putamen FC						
Orbitofrontal gyrus	L	24	-6	39	-9	-3.490
PD-NA > HC						
Left putamen FC						
Orbitofrontal gyrus	R	107	6	63	-21	4.773
Right putamen FC						
Orbitofrontal gyrus	L	16	-18	60	-18	3.954
Middle occipital gyrus	L	139	-27	-102	-9	4.113
Paracentral lobule	R	94	6	-27	75	3.922
PD-NA < HC						
Left putamen FC						
Anterior cingulate gyrus	L	185	-9	36	3	-3.962
Middle frontal gyrus	L	136	-33	42	15	-3.903
Postcentral gyrus	R	117	27	-39	48	-4.222
Right putamen FC						
Cerebellum	R	23	12	-69	-36	-3.271
Middle frontal gyrus	L	18	-15	54	3	-3.012
Middle cingulate gyrus	L	18	-3	0	33	-3.501

Post hoc two sample t-tests with age, gender, education, HAMD scores and grey matter volume as covariates were performed to test differences between groups on the putamen FC. Note: FC, functional connectivity; MNI, Montreal Neurological Institute; HC, healthy controls; PD-A, Parkinson's disease patients with anxiety; PD-NA, Parkinson's disease patients without anxiety; Results are displayed at *p* < 0.05 corrected by AlphaSim.

PD-A subjects showed following differences with the healthy control subjects: (1) reduced left putamen FC with left anterior cingulate; (2) reduced right putamen FC with left orbitofrontal gyrus and (3) increased right putamen FC with paracentral lobule (Table 2 and Figure 1).

The PD-NA subjects showed the following differences with the healthy control subjects: (1) decreased left putamen FC with left anterior cingulated gyrus, left middle frontal gyrus and right postcentral cortex; (2) increased left putamen FC with right orbitofrontal gyrus; (3) reduced right putamen FC with right cerebellum, left middle frontal gyrus and left middle cingulated gyrus; and (4) increased right putamen FC with left orbitofrontal gyrus, left middle occipital gyrus and right paracentral lobule (Table 2 and Figure 1).

**Figure 2:**
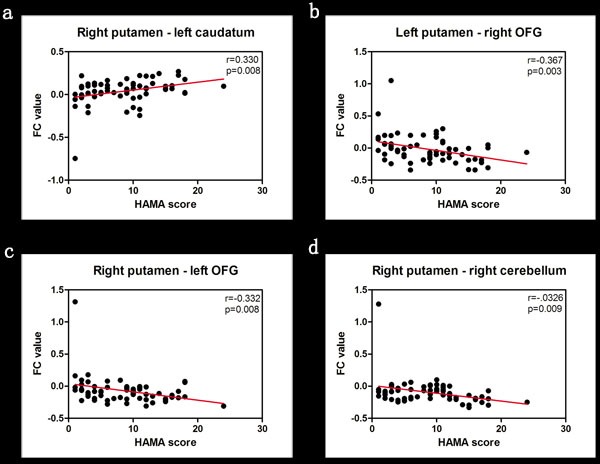
Correlation analysis between HAMA scores and FC values of brain regions in the study groups The positive and negative correlations in PD-A patients based on the Pearson correlation analysis between mean FC values and HAMA scores are shown. *P* < 0.01 was considered statistically significant. Note: HAMA, Hamilton Anxiety Rating Scale; FC, functional connectivity; OFG, orbitofrontal gyrus.

Figure 2 shows Pearson correlation analysis of HAMA scores and FC scores corresponding to differences between PD-A and PD-NA groups. The analysis revealed the following: (1) positive correlation between right putamen FC with left caudatum (*r* = 0.330, *p* = 0.008); (2) negative correlation between left putamen FC with right orbitofrontal gyrus (*r* = -0.367, *p* = 0.003) and (3) negative correlation between right putamen FC with left orbitofrontal gyrus (*r* = -0.332, *p* = 0.008) and right cerebellum (*r* = -0.326, *p* = 0.009).

## DISCUSSION

In the present study, we compared the FC alterations in the putamen region of the brain in Parkinson's patients with anxiety disorder to Parkinson's patients without anxiety and healthy controls. Rs-fMRI analysis revealed significant alterations in FC in the PD-A patients compared to the PD-NA and HC groups. The PD-A patients showed decreased left putamen FC with right orbitofrontal gyrus and right putamen FC with left orbitofrontal gyrus, right cerebellum and right precuneus. On the other hand, there was increased right putamen FC with right insula, left temporal pole, left middle occipital gyrus, left caudatum and right middle cingulate gyrus compared to PD patients without anxiety. This suggested abnormal putamen FC in the cortical and subcortical regions in PD-A patients.

The HAMA scores showed positive correlation between right putamen FC and left caudatum, but negative correlation for left putamen FC with right orbitofrontal gyrus and right putamen FC with left orbitofrontal gyrus and right cerebellum. These results demonstrate that anxiety in PD patients correlates with the aberrant putamen FC in them, especially with the caudatum, orbitofrontal gyrus and cerebellum.

Neuroimaging studies with ^123^I-FP-CIT SPECT, SPECT with TRODAT-1 or PET have explored anxiety severity in PD patients by assessing dopamine transporter (DAT) availability or density [6–9, 25, 26]. Most studies observed an inverse correlation between DAT availability in the putamen with the severity of anxiety in PD [6, 7, 9]. However, Ceravolo *et al*. showed that increased striatal DAT density was associated with mild anxiety symptoms in PD patients [8]. The PD rat model depleted of striatal dopamine showed increased anxiety [27, 28], which improved upon L-DOPA treatment [29, 30]. Furthermore, social anxiety disorder, which is common in PD patients was associated with dopaminergic-mediated striatal circuits [31] and sustained suppression of DA receptor activity [32]. These findings suggest a relationship between anxiety and dopaminergic dysfunction in PD patients. Therefore, we focused on interactions of the bilateral putamen with other brain areas in the present study.

Our results show increased putamen FC with caudate in PD-A relative to PD-NA patients. Although striatal dysfunction is typically related to motor impairment in PD [33], it is implicated in emotional processing [34], thereby suggesting a common pathophysiological mechanism. Neuroimaging by [^18^F] fluorodeoxyglucose PET (FDG-PET) showed that anxiety symptoms in PD were inversely associated with DAT availability in the caudatum or decreased caudate metabolism [35]. Therefore, enhanced putamen FC and caudatum suggested emotional processing during anxiety in PD patients.

We also observed reduced bilateral putamen FC with orbitofrontal gyrus in PD-A compared to PD-NA patients. Previous studies in non-PD patients demonstrated that orbitofrontal gyrus as the site for emotion processing. Milad *et al.* showed the role of medial orbitofrontal gyrus in conditioned fear response based on various animal studies and human neuroimaging analysis [36]. Patients with agoraphobia, which is a panic disorder showed decreased gray matter volume in their left medial orbitofrontal gyrus compared to healthy controls [37]. Moreover, patients with impaired orbitofrontal gyrus showed mental disturbances such as impulsive, socially inappropriate behavior and emotional changes compared to control subjects [38]. This suggested the role of impaired orbitofrontal gyrus in anxiety disorders. Additionally, a T1-weighted MRI study in patients with early PD showed that focal orbitofrontal gyrus atrophy led to anxiety disorder [39]. Topographically, orbitofrontal gyrus projects into regions of the striatum [40] and the frontostriatal pathway has been implicated in anxiety [41]. Based on these considerations, attenuated connections between orbitofrontal gyrus and putamen suggest abnormal frontostriatal pathway, which contributes to anxiety disorder in PD.

There was also reduced FC between cerebellum and putamen in PD-A relative to PD-NA subjects, thereby negatively correlating with anxiety severity. Clinical, experimental and neuroimaging studies indicate that the cerebellum is involved in neural processes beyond the motor domain [42, 43]. For example, electrical stimulation of the human cerebellum produces anxiety and fear [44]. The synaptic connections between parallel fibers and Purkinje cells of cerebellum are involved in the learned fear and their long-term potentiation contributes to fear memory [45]. In functional brain imaging studies with PET, increased blood flow was observed in the left cerebellar hemisphere during fear conditioning [46]. Therefore, both human and animal studies have shown that cerebellum contributes to fear-associated learning. Recently, a meta-analysis of neuroimaging studies on functional topography in the human cerebellum showed involvement of the cerebellar posterior lobe in cognitive/emotional function [42]. This is in agreement with our findings of decreased cerebellum FC, especially in the posterior lobe. Consequently, decreased communication between cerebellum and putamen plays a role in anxiety disorders observed in PD patients.

This study has several limitations. Firstly, our sample size was relatively small and therefore may not have detected exact neural substrates of PD-related anxiety disorder. Secondly, anxiety and depression overlap in PD patients with similar symptoms and pathophysiology [20, 47]. Hence, patients with clinically distinct depressive symptoms were excluded from our study. However, HAMD scores still showed significant differences between study subject groups and therefore, FC was analyzed. Besides, age and gender were significantly different among groups. Many studies have shown correlation between PD-related anxiety disorder with age and gender [2, 48]. Therefore, these were analyzed as covariates. Finally, although the present study defined important regions of interest that are responsible for anxiety in PD, it did not represent complete FC analysis. Therefore, further comprehensive studies are needed to identify other anxiety related networks that were not analyzed by our study.

In conclusion, our study showed that anxiety in PD was associated with altered FC network between putamen and other regions of the brain, especially caudatum, orbitofrontal gyrus and cerebellum.

## References

[1] Broen MP, Narayen NE, Kuijf ML, Dissanayaka NN, Leentjens AF (2016). Prevalence of anxiety in Parkinson's disease: A systematic review and meta-analysis. Mov Disord.

[2] Dissanayaka NN, Sellbach A, Matheson S, O’Sullivan JD, Silburn PA, Byrne GJ, Marsh R, Mellick GD (2010). Anxiety disorders in Parkinson's disease: prevalence and risk factors. Mov Disord.

[3] Pontone GM, Williams JR, Anderson KE, Chase G, Goldstein SA, Grill S, Hirsch ES, Lehmann S, Little JT, Margolis RL, Rabins PV, Weiss HD, Marsh L (2009). Prevalence of anxiety disorders and anxiety subtypes in patients with Parkinson's disease. Mov Disord.

[4] Wen MC, Chan LL, Tan LC, Tan EK (2016). Depression, anxiety, and apathy in Parkinson's disease: insights from neuroimaging studies. Eur J Neurol.

[5] Weingarten CP, Sundman MH, Hickey P, Chen NK (2015). Neuroimaging of Parkinson's disease: Expanding views. Neurosci Biobehav Rev.

[6] Weintraub D, Newberg AB, Cary MS, Siderowf AD, Moberg PJ, Kleiner-Fisman G, Duda JE, Stern MB, Mozley D, Katz IR (2005). Striatal dopamine transporter imaging correlates with anxiety and depression symptoms in Parkinson's disease. J Nucl Med.

[7] Erro R, Pappata S, Amboni M, Vicidomini C, Longo K, Santangelo G, Picillo M, Vitale C, Moccia M, Giordano F, Brunetti A, Pellecchia MT, Salvatore M (2012). Anxiety is associated with striatal dopamine transporter availability in newly diagnosed untreated Parkinson's disease patients. Parkinsonism Relat Disord.

[8] Ceravolo R, Frosini D, Poletti M, Kiferle L, Pagni C, Mazzucchi S, Volterrani D, Bonuccelli U (2013). Mild affective symptoms in de novo Parkinson's disease patients: relationship with dopaminergic dysfunction. Eur J Neurol.

[9] Moriyama TS, Felicio AC, Chagas MH, Tardelli VS, Ferraz HB, Tumas V, Amaro-Junior E, Andrade LA, Crippa JA, Bressan RA (2011). Increased dopamine transporter density in Parkinson's disease patients with Social Anxiety Disorder. J Neurol Sci.

[10] Fox MD, Raichle ME (2007). Spontaneous fluctuations in brain activity observed with functional magnetic resonance imaging. Nat Rev Neurosci.

[11] Rosazza C, Minati L (2011). Resting-state brain networks: literature review and clinical applications. Neurol Sci.

[12] Lee MH, Smyser CD, Shimony JS (2013). Resting-state fMRI: a review of methods and clinical applications. AJNR Am J Neuroradiol.

[13] Wang M, Jiang S, Yuan Y, Zhang L, Ding J, Wang J, Zhang J, Zhang K, Wang J (2016). Alterations of functional and structural connectivity of freezing of gait in Parkinson's disease. J Neurol.

[14] Hu X, Song X, Yuan Y, Li E, Liu J, Liu W, Liu Y (2015). Abnormal FC of the amygdala is associated with depression in Parkinson's disease. Mov Disord.

[15] Su M, Wang S, Fang W, Zhu Y, Li R, Sheng K, Zou D, Han Y, Wang X, Cheng O (2015). Alterations in the limbic/paralimbic cortices of Parkinson's disease patients with hyposmia under resting-state functional MRI by regional homogeneity and FC analysis. Parkinsonism Relat Disord.

[16] Zhang JJ, Ding J, Li JY, Wang M, Yuan YS, Zhang L, Jiang SM, Wang XX, Zhu L, Zhang KZ (2017). Abnormal Resting-State Neural Activity and Connectivity of Fatigue in Parkinson's Disease. CNS Neurosci Ther.

[17] Hughes AJ, Daniel SE, Kilford L, Lees AJ (1992). Accuracy of clinical diagnosis of idiopathic Parkinson's disease: a clinico-pathological study of 100 cases. J Neurol Neurosurg Psychiatry.

[18] Leentjens AF, Dujardin K, Marsh L, Richard IH, Starkstein SE, Martinez-Martin P (2011). Anxiety rating scales in Parkinson's disease: a validation study of the Hamilton anxiety rating scale, the Beck anxiety inventory, and the hospital anxiety and depression scale. Mov Disord.

[19] Stefanova E, Ziropadja L, Petrovic M, Stojkovic T, Kostic V (2013). Screening for anxiety symptoms in Parkinson disease: a cross-sectional study. J Geriatr Psychiatry Neurol.

[20] Jiang SM, Yuan YS, Tong Q, Zhang L, Xu QR, Ding J, Zhang KZ (2015). The association between clinically relevant anxiety and other non-motor symptoms in Parkinson's disease. Neurol Sci.

[21] Tomlinson CL, Stowe R, Patel S, Rick C, Gray R, Clarke CE (2010). Systematic review of levodopa dose equivalency reporting in Parkinson's disease. Mov Disord.

[22] Chao-Gan Y, Yu-Feng Z (2010). DPARSF: A MATLAB Toolbox for “Pipeline” Data Analysis of Resting-State fMRI. Front Syst Neurosci.

[23] Goto M, Abe O, Aoki S, Hayashi N, Miyati T, Takao H, Iwatsubo T, Yamashita F, Matsuda H, Mori H, Kunimatsu A, Ino K, Yano K (2013). Diffeomorphic Anatomical Registration Through Exponentiated Lie Algebra provides reduced effect of scanner for cortex volumetry with atlas-based method in healthy subjects. Neuroradiology.

[24] Power JD, Barnes KA, Snyder AZ, Schlaggar BL, Petersen SE (2012). Spurious but systematic correlations in FC MRI networks arise from subject motion. Neuroimage.

[25] Di Giuda D, Camardese G, Bentivoglio AR, Cocciolillo F, Guidubaldi A, Pucci L, Bruno I, Janiri L, Giordano A, Fasano A (2012). Dopaminergic dysfunction and psychiatric symptoms in movement disorders: a 123I-FP-CIT SPECT study. Eur J Nucl Med Mol Imaging.

[26] Remy P, Doder M, Lees A, Turjanski N, Brooks D (2005). Depression in Parkinson's disease: loss of dopamine and noradrenaline innervation in the limbic system. Brain.

[27] Tadaiesky MT, Dombrowski PA, Figueiredo CP, Cargnin-Ferreira E, Da Cunha C, Takahashi RN (2008). Emotional, cognitive and neurochemical alterations in a premotor stage model of Parkinson's disease. Neuroscience.

[28] Taylor TN, Caudle WM, Shepherd KR, Noorian A, Jackson CR, Iuvone PM, Weinshenker D, Greene JG, Miller GW (2009). Nonmotor symptoms of Parkinson's disease revealed in an animal model with reduced monoamine storage capacity. J Neurosci.

[29] Funkiewiez A, Ardouin C, Cools R, Krack P, Fraix V, Batir A, Chabardes S, Benabid AL, Robbins TW, Pollak P (2006). Effects of levodopa and subthalamic nucleus stimulation on cognitive and affective functioning in Parkinson's disease. Mov Disord.

[30] Stacy MA, Murck H, Kroenke K (2010). Responsiveness of motor and nonmotor symptoms of Parkinson disease to dopaminergic therapy. Prog Neuropsychopharmacol Biol Psychiatry.

[31] Stein DJ, Westenberg HG, Liebowitz MR (2002). Social anxiety disorder and generalized anxiety disorder: serotonergic and dopaminergic neurocircuitry. J Clin Psychiatry.

[32] Schneier FR, Liebowitz MR, Abi-Dargham A, Zea-Ponce Y, Lin SH, Laruelle M (2000). Low dopamine D(2) receptor binding potential in social phobia. Am J Psychiatry.

[33] Pavese N (2012). PET studies in Parkinson's disease motor and cognitive dysfunction. Parkinsonism Relat Disord.

[34] Sander D, Grandjean D, Pourtois G, Schwartz S, Seghier ML, Scherer KR, Vuilleumier P (2005). Emotion and attention interactions in social cognition: brain regions involved in processing anger prosody. Neuroimage.

[35] Huang C, Ravdin LD, Nirenberg MJ, Piboolnurak P, Severt L, Maniscalco JS, Solnes L, Dorfman BJ, Henchcliffe C (2013). Neuroimaging markers of motor and nonmotor features of Parkinson's disease: an 18f fluorodeoxyglucose positron emission computed tomography study. Dement Geriatr Cogn Disord.

[36] Milad MR, Rauch SL (2007). The role of the orbitofrontal cortex in anxiety disorders. Ann N Y Acad Sci.

[37] Na KS, Ham BJ, Lee MS, Kim L, Kim YK, Lee HJ, Yoon HK (2013). Decreased gray matter volume of the medial orbitofrontal cortex in panic disorder with agoraphobia: a preliminary study. Prog Neuropsychopharmacol Biol Psychiatry.

[38] Berlin HA, Rolls ET, Kischka U (2004). Impulsivity, time perception, emotion and reinforcement sensitivity in patients with orbitofrontal cortex lesions. Brain.

[39] Tinaz S, Courtney MG, Stern CE (2011). Focal cortical and subcortical atrophy in early Parkinson's disease. Mov Disord.

[40] Sgambato-Faure V, Worbe Y, Epinat J, Feger J, Tremblay L (2016). Cortico-basal ganglia circuits involved in different motivation disorders in non-human primates. Brain Struct Funct.

[41] de Visser L, Baars AM, Lavrijsen M, van der Weerd CM, van den Bos R (2011). Decision-making performance is related to levels of anxiety and differential recruitment of frontostriatal areas in male rats. Neuroscience.

[42] Stoodley CJ, Schmahmann JD (2009). Functional topography in the human cerebellum: a meta-analysis of neuroimaging studies. Neuroimage.

[43] Sacchetti B, Scelfo B, Strata P (2005). The cerebellum: synaptic changes and fear conditioning. Neuroscientist.

[44] Nashold BS, Wilson WP, Slaughter DG (1969). Sensations evoked by stimulation in the midbrain of man. J Neurosurg.

[45] Sacchetti B, Scelfo B, Tempia F, Strata P (2004). Long-term synaptic changes induced in the cerebellar cortex by fear conditioning. Neuron.

[46] Fischer H, Andersson JL, Furmark T, Fredrikson M (2000). Fear conditioning and brain activity: a positron emission tomography study in humans. Behav Neurosci.

[47] Leentjens AF, Dujardin K, Marsh L, Martinez-Martin P, Richard IH, Starkstein SE (2011). Symptomatology and markers of anxiety disorders in Parkinson's disease: a cross-sectional study. Mov Disord.

[48] Liu R, Umbach DM, Peddada SD, Xu Z, Troster AI, Huang X, Chen H (2015). Potential sex differences in nonmotor symptoms in early drug-naive Parkinson disease. Neurology.

